# Interest in Cardiothoracic Surgery Among the American College of Osteopathic Surgeons’ Medical Students

**DOI:** 10.7759/cureus.65672

**Published:** 2024-07-29

**Authors:** Andrew D Vogel, Austin B Wynn, Megan C Richards, Michelle Sindoni, Zachary Brennan, Caleb L Hamilton, Juan J Gallegos, Tyler J Wallen

**Affiliations:** 1 Research, Alabama College of Osteopathic Medicine, Dothan, USA; 2 Cardiac Surgery, Smidt Heart Institute, Cedars-Sinai Medical Center, Los Angeles, USA; 3 Health and Movement Sciences, School of Health and Movement Sciences, Missouri Baptist University, St. Louis, USA; 4 Cardiothoracic Surgery, Huntsville Hospital Health System, Huntsville, USA; 5 Cardiovascular Surgery, Geisinger Commonwealth School of Medicine, Wilkes-Barre, USA

**Keywords:** medical education, interest groups, surgical education, medical students, cardiothoracic surgery

## Abstract

Background: As more integrated cardiothoracic (CT) surgical residency programs are developed, there is increased interest in factors influencing specialty selection during undergraduate medical education. This study aimed to nationally assess interests and perceptions of CT surgery from medical students pursuing surgery and factors influencing such interests.

Methods: Active members of the American College of Osteopathic Surgeons - Medical Student Section were invited to complete an original survey. Means and 95% confidence intervals were calculated and graphed for questions using Likert scale responses. The comparison of mean responses for students in preclinical versus clinical years was assessed by a Kruskal-Wallis non-parametric ANOVA. Differences between response proportions were assessed via Bonferroni Comparison of Column Proportions.

Results: There were 306 surveys completed. Interest in CT surgery was indicated by 10.24% of respondents with preclinical students comprising 84.3% of those responses. Most students interested in CT surgery experienced certain factors including clinical exposure (78.4%), shadowing (81.8%), volunteering (57.1%), and significant personal/life events (86.2%) before medical school. Preclinical students noted exposure to CT surgery during preclinical years would further increase their interest when compared to clinical students (μ=4.12 versus μ=3.51, P<0.000). Importantly, clinical students feel significantly less supported by their school to pursue CT surgery compared to preclinical students (μ=2.45 versus μ=3.40, P<0.000).

Conclusions:All factors establishing interest in CT surgery occurred before students entered medical school and during preclinical years. While there are negative perceptions associated with CT surgery, these may be ameliorated with increased support and resources for CT surgery during the preclinical years.

## Introduction

Cardiothoracic (CT) surgery is a surgical subspecialty that has struggled to recruit medical students to its field [1-5]. With CT surgical residency programs expanding, interest in factors influencing specialty selection during and before undergraduate medical education is growing [1,2,5-8]. Previous studies assessing CT surgery interest have been completed at large academic institutions and osteopathic institutions [1,5,7,9,10]. These demonstrated that factors decreasing interest in CT surgery include work/life balance, the personality of CT surgeons, and lack of family time associated with the field [5,7]. Our studies unveiled negative factors associated with CT surgery which may be ameliorated with more exposure to the field earlier in a student’s career [5]. In addition, most experiences and events that draw students to CT surgery occurred before entering medical school and during their preclinical years of medical school [5,11]. While all these studies are insightful, they have only been completed at an institutional level. Although a previous trial surveyed members of the Thoracic Surgery Medical Student Association (TSMA), an association for students interested in CT surgery, there has not yet been a national assessment of medical students interested in surgery and other surgical subspecialties to assess their interest and perceptions of CT surgery [12].

This is the final study in a trilogy that has assessed medical students' interest in CT surgery from one institution at the beginning of the academic year, determined the impact of a CT surgery interest committee on preclinical students' interest in the field, and, finally, nationally assessed medical student's interest in CT surgery via the American College of Osteopathic Surgeons - Medical Student Section (ACOS-MSS). The aim of this study was to assess factors affecting students’ pursuit of CT surgery, factors discouraging the pursuit of a career in CT surgery, experiences influencing students’ interest in CT surgery and when they occurred, cognizance of CT surgery resources, perceptions of CT surgery, and preclinical and clinical exposure and support for surgery.

## Materials and methods

Ethics

This project was classified as exempt by the Institutional Review Board of the Alabama College of Osteopathic Medicine. All students took the anonymous survey voluntarily and gave informed consent. Survey results were reported in aggregate without identifying information.

Survey population

The ACOS-MSS is the official student section of the ACOS. The purpose of ACOS-MSS is to represent and educate future osteopathic surgeons through local chapters of ACOS-MSS at colleges of osteopathic medicine. There are currently 44 osteopathic medical schools that have associated local ACOS-MSS chapters.

Survey instrument and recruitment

A de novo online survey instrument was constructed with input from past literature [5,7,10,13]. The survey instrument questions are provided as Supplemental Material 1 (see Appendix). The survey was created with an online survey platform and distributed to all medical students via ACOS-MSS E-mail list-serves, the X platform, and in person at the ACOS-MSS spring conference. The survey was conducted over the 2022-2023 academic year starting in October 2023 and ending in April 2024.

Statistical analysis

Medical students were invited to complete an original online survey via Qualtrics XM (Qualtrics, Provo, Utah). About 369 survey responses were initiated, but 40 were excluded from the analysis for not participating beyond the initial question of consent. Additional missing responses for individual questions were excluded from the analysis. Statistical analysis of data was performed using IBM SPSS Statistics for Windows, Version 28 (Released 2021; IBM Corp., Armonk, New York, United States). Chi-square analysis (two-tailed), with a Bonferroni comparison of column proportions, was used to compare the difference in response probability between pre-clinical students, as defined by first and second-year osteopathic medical students, and clinical students, as defined by third and fourth-year osteopathic medical students. The map representing the location and frequency of responses from each college of medicine was generated using Google Sheets (November 2023, Google, Mountainview, United States).

Questions assessing participants’ Likert scale (1-5) ranked responses were also utilized. Mean values and standard error were calculated for the total population and the clinical and pre-clinical subpopulations. Differences between mean rank values between clinical and pre-clinical students were compared with a Kruskal-Wallis non-parametric ANOVA (two-tailed) and significance was determined by a p <0.05. GraphPad Prism version 10.1.2 for Windows (GraphPad, San Diego, United States) was used for the generation of graphs.

## Results

Demographics

**Figure 1:**
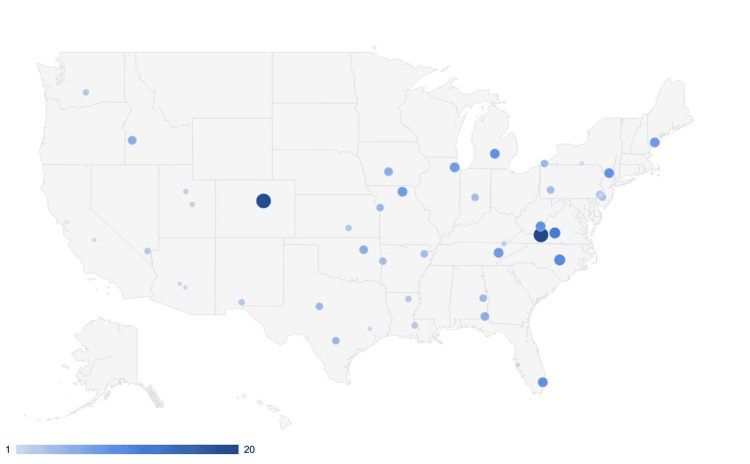
National map of survey respondents' location The geographic location of medical schools attended is depicted by blue circles in the United States map. The increasing size and darkness of the blue circle correspond with the increased frequency of responses.

The survey was completed by 306 students. Female students comprised 55.9% of respondents and males comprised 43.8%. The greatest number of participants were 24 to 26 years old, at 52.1% of total respondents. Preclinical students represented 67.7% of survey responses. Nontraditional students who did not start medical school immediately after completing their undergraduate program comprised 82.4% of respondents. Figure 1 demonstrates a geographic representation of respondents with most residing in the eastern United States.

Interest in CT surgery

**Figure 2:**
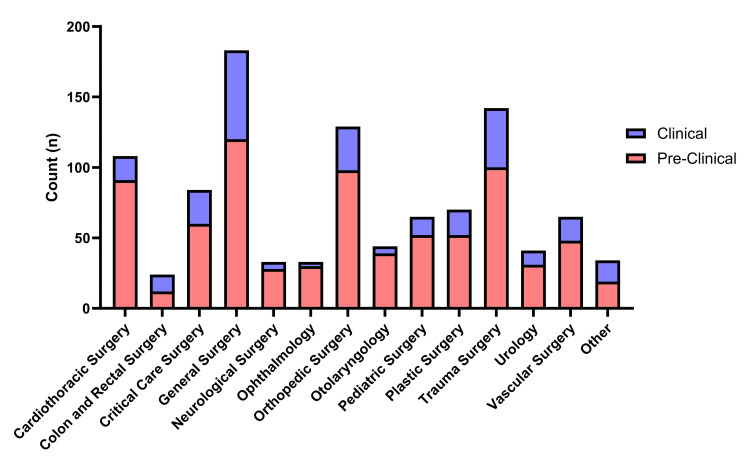
Interest in surgical subspecialties The number of pre-clinical (red) and clinical (blue) students is depicted in the bar graph for each surgical subspecialty. The total response counts for each subspecialty are indicated by the stacked bars, with the counts of each group (clinical and preclinical) depicted by the individual color segment of the bars.

Interest in CT surgery represented 10.24% of responses, 84.3% of which were preclinical students. When asked to select specialties of interest, 11.67% of preclinical students were interested in CT surgery compared to 6.18% of clinical students (P=0.016). Surgery subspecialties with a greater than 10% response rate of the total population included CT surgery (10.24%), orthopedic surgery (12.23%), trauma surgery (13.46%), and general surgery (17.35%) (Figure 2).

Factors deterring students from CT surgery

**Figure 3:**
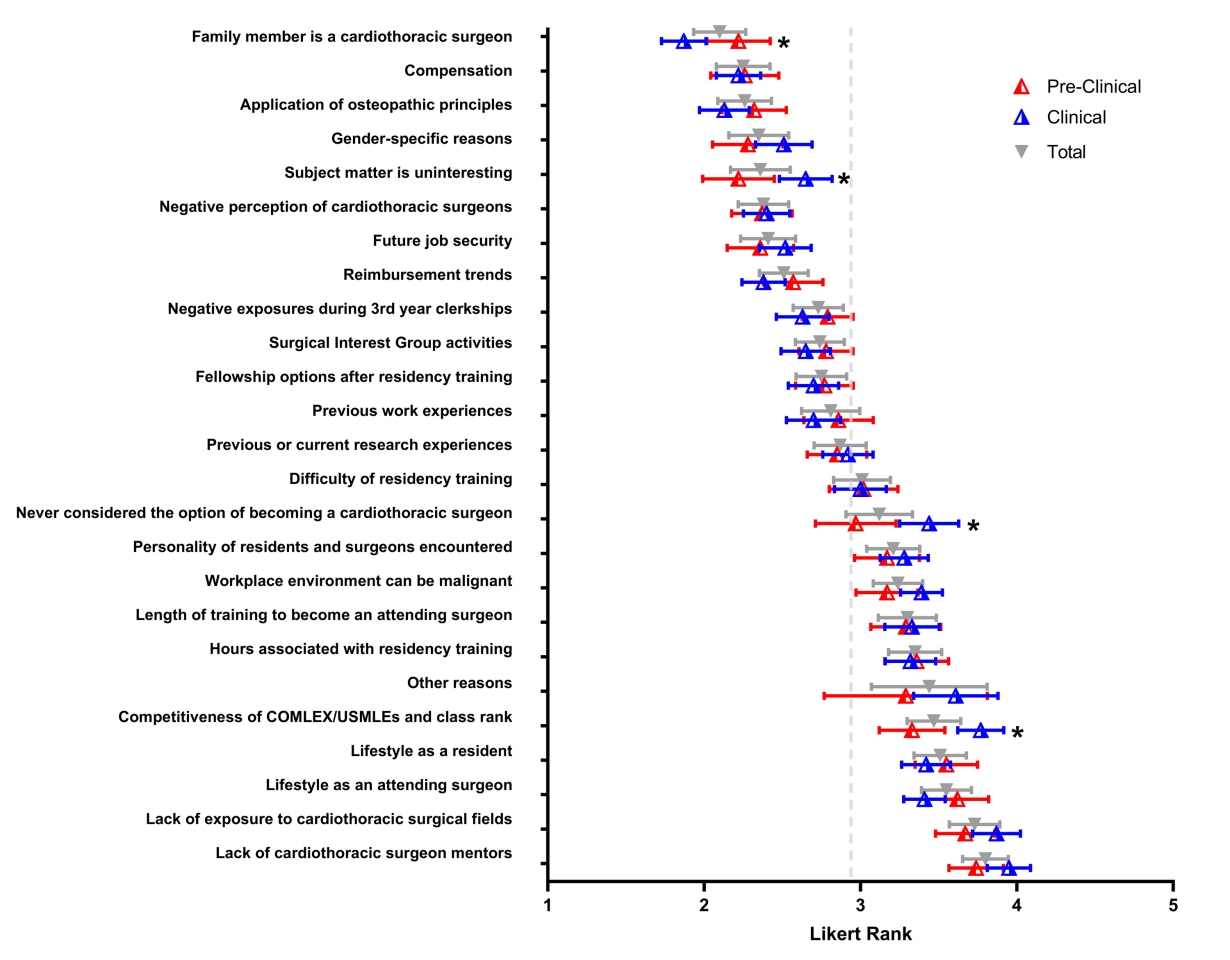
Factors influencing the decision not to pursue cardiothoracic surgery Response rankings for the relative impact of factors influencing participants’ decision not to pursue cardiothoracic surgery were analyzed. Total mean values are depicted by the gray triangle in the scatter plot, with error bars representing 95% confidence intervals. Mean rank responses of pre-clinical (red) and clinical (blue) mean values are depicted with 95% confidence intervals. The average mean value of responses for all questions is depicted by the gray vertical dashed line. Median rank values were assessed with a Kruskal-Wallis non-parametric ANOVA. Significance between the clinical and pre-clinical groups is denoted by an asterisk(*) when P < 0.05 (two-tailed).

Participants were asked to identify their level of agreement with multiple factors that influenced their decision to not pursue CT surgery. On a Likert scale (1 - strongly disagree, 2 - somewhat disagree, 3 - neither agree nor disagree, 4 - somewhat agree, and 5 - strongly agree), the reasons to not pursue CT surgery with highest agreement ratings were lifestyle as a resident, lifestyle as an attending surgeon, lack of exposure to CT surgical fields, and lack of CT surgeon mentors, with the mean values of all students at 3.51, 3.55, 3.73, and 3.80, respectively (Figure 3). Clinical students selected the competitiveness of board scores and class rank as a more significant factor than preclinical students for not pursuing CT surgery (µ=3.77 versus µ=3.33, P=0.012). Another significant difference found was clinical students agreeing the factor of never considering the option of becoming a CT surgeon deterred them more so than preclinical students (µ=3.44 versus µ=2.97, P=0.028).

Factors developing and continuing interest in CT surgery

**Figure 4:**
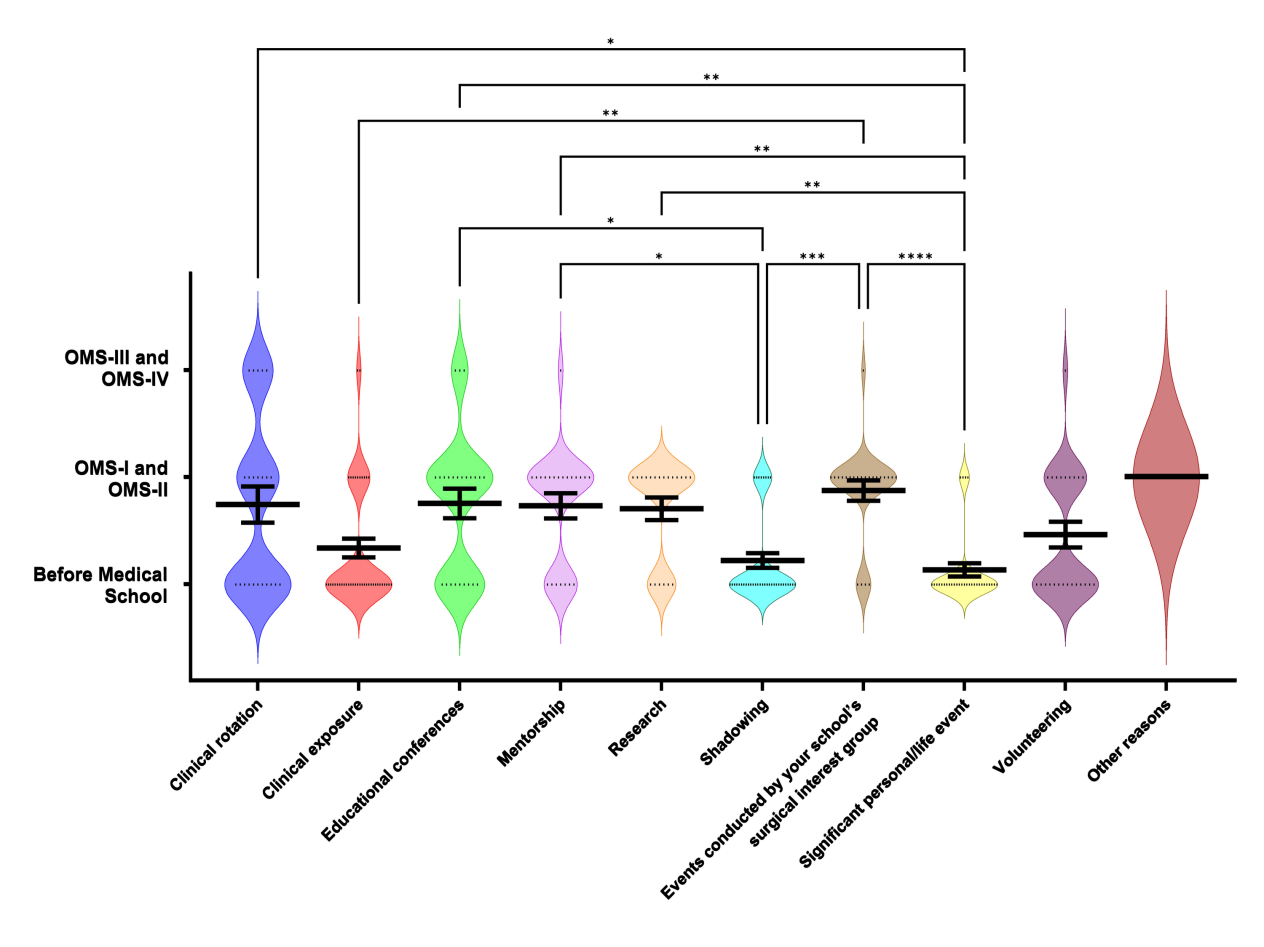
Factors contributing to the decision to pursue cardiothoracic surgery Factors contributing to survey participants’ interest in pursuing cardiothoracic surgery were assessed for the respective time relative to student’s medical school training (before medical school, OMS- I & II, or OMS- III & IV). Violin plots are used to depict the response frequencies for each factor at each time point, with black bars indicating the mean (central line) and standard error of the mean (outer lines). The occurrence relative to the student’s medical school training was ranked categorically, and median rank values were compared using Kruskal-Wallis ANOVA. Significance is denoted by asterisk(s) when significant (two-tailed; *: P < 0.05, **: P < 0.01, ***: P < 0.001, ****: P < 0.0001).

In response to factors developing and continuing interest in CT surgery, students selected multiple reasons for interest in CT surgery and selected if that reason occurred before medical school, during osteopathic medical student (OMS)-I/II, or during OMS-III/IV which corresponded to a number value of one, two, or three, respectively (Figure 4). The most selected factors were clinical exposure (16.97%) and shadowing (15.14%). Of the students who selected clinical exposure, mentorship, shadowing, significant personal/life events, and volunteering, 78.4%, 31.6%, 81.8%, 86.2%, and 57.1% stated that it occurred before medical school, respectively. Students selected research, educational conferences, and events conducted by surgical interest groups, 66.7%, 40.0%, and 80.0%, respectively, occurring most often during OMS-I/II. Significant differences in occurrence were seen in clinical exposure, shadowing, and significant personal/life events. Clinical exposure developed and continued interest significantly earlier than events conducted by a school’s interest groups (μ=1.24 versus μ=1.87, P=0.0048). Shadowing garnered interest significantly sooner than events conducted by a school’s interest group (μ=1.18 versus μ=1.80, P=0.0004). Significant personal/life events (μ=1.14) developed and continued interest significantly sooner than educational conferences (μ=1.70), mentorship (μ=1.74), research (μ=1.67), and events conducted by a school’s interest group (μ=1.80, P<0.01). Most respondents stated that clinical rotations started or continued interest closer to OMS-I/II. This value may have been skewed and the question considered not clear since clinical rotations cannot occur before OMS-III/IV.

Knowledge and awareness of resources in CT surgery

**Figure 5:**
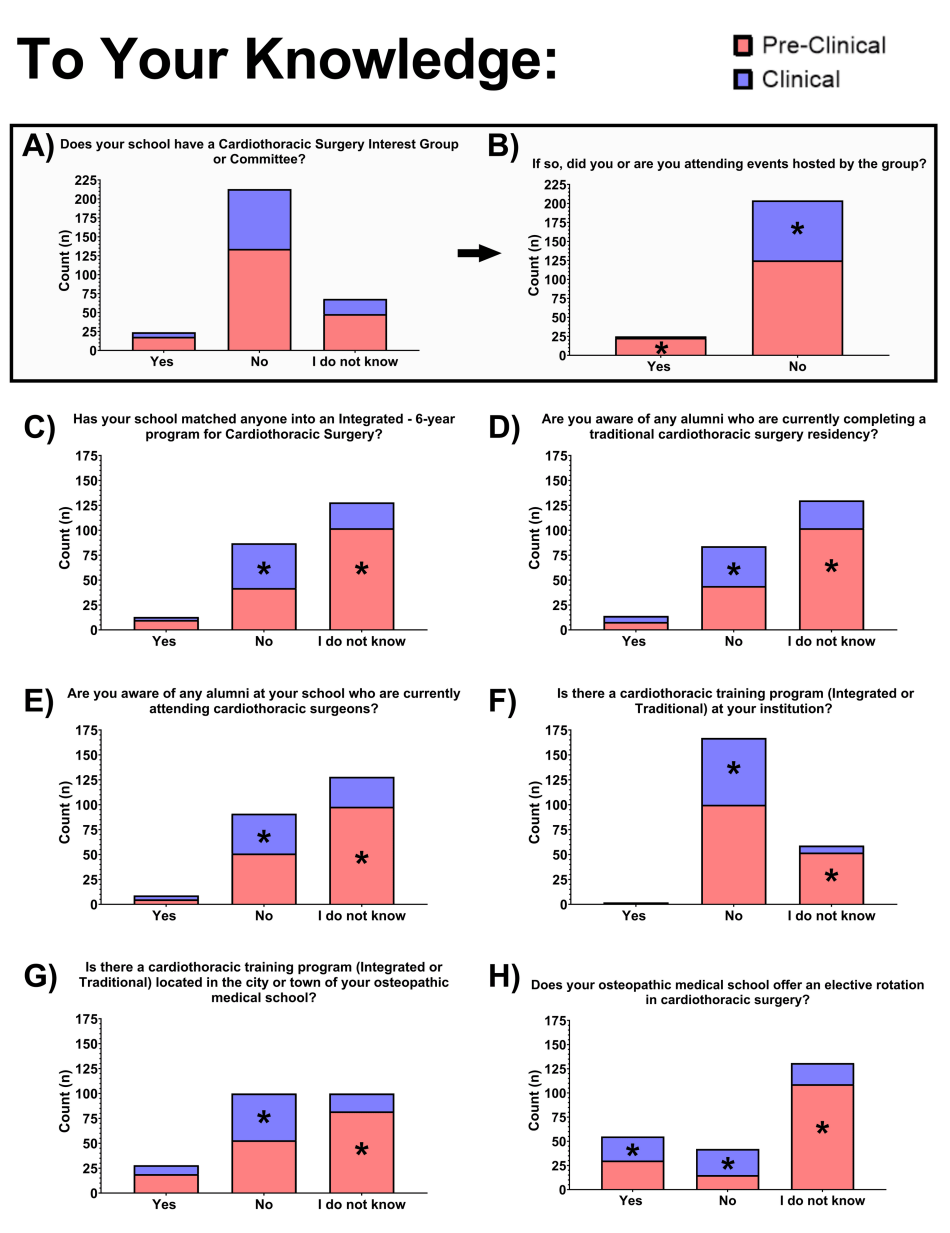
Knowledge of cardiothoracic surgery school and training opportunities Survey responses regarding participants’ knowledge of cardiovascular surgery opportunities and successes at their institution, and training opportunities, were collected. Total response numbers are depicted in stacked bar graphs, with the number of responses from pre-clinical participants shown in the red bar segments, and clinical participant responses shown in blue. Comparisons of the proportion of responses between pre-clinical and clinical students were assessed using a Bonferroni comparison of column proportions. Significance is denoted by an asterisk (*) in the group (pre-clinical or clinical) with a significantly greater proportion of “yes,” “no,” or “I do not know,” responses when P < 0.05 (two-tailed).

Questions regarding cognizance of different resources for the pursuit of CT surgery were given as yes, no, or I am not sure options (Figure 5). A significantly greater proportion of preclinical students indicated they were unsure if anyone from their school matched into an integrated six-year program for CT surgery (66.23%, P<0.000), if any alumni were completing a traditional CT surgery residency (66.23%, P<0.000), or if any alumni were attending CT surgeons (63.64%, P<0.003) compared to clinical students.

Students were then asked about CT surgery training programs within their institution or local area. When asked if there were CT training programs at their institution, 73.25% of students said no. About 53.25% of preclinical students were unsure if CT training programs were in the city or town of their osteopathic institution. A significantly greater proportion of clinical students stated that there was not a CT training program (63.51%, P=0.000) compared to preclinical students.

The last question of the survey asked students if electives for CT surgery were offered within their osteopathic institutions. About 57.46% of students were unsure if their osteopathic institution offered elective rotations in CT surgery. Most students also stated that their school does not have a CT surgery interest group or committee (69.9%).

Perceptions of CT surgery, exposure, and support

**Figure 6:**
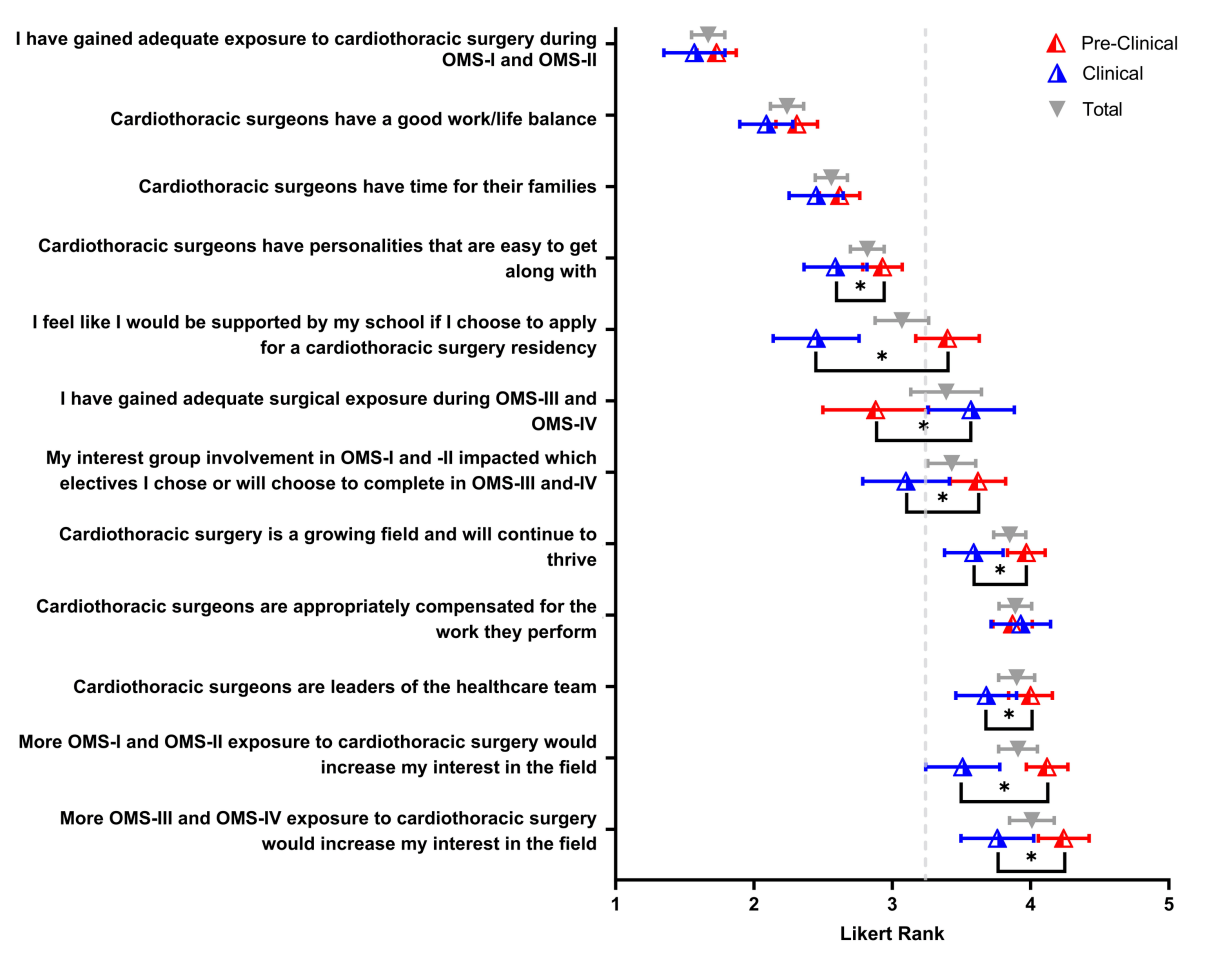
Perceptions involving cardiothoracic surgery training and careers Participants’ Likert-ranked responses to their perceptions regarding their cardiothoracic surgery training experiences, and perceptions regarding careers in cardiothoracic surgery, are depicted in the scatter plot. Total values are represented by the gray triangle, with pre-clinical mean rank values (red), and clinical mean rank values (blue) depicted. Error bars represent 95% confidence intervals. The gray vertical dashed line is indicative of the average cumulative response for all questions. Comparisons of median rank values between pre-clinical and clinical students were assessed with a Kruskal-Wallis ANOVA. Significance is denoted by an asterisk (*) when P < 0.05 (two-tailed).

Multiple statements were assessed by students regarding their perceptions of CT surgeons, how they perceive CT surgery overall, and insight into their exposure to CT surgery through their institution (Figure 6). Factors associated with CT surgery with low agreement rankings included work/life balance, the personality of CT surgeons that are easy to get along with, and having time with their families, with overall means of 2.24, 2.82, and 2.56, respectively. Preclinical student responses were significantly different than clinical student responses to the perception of the personalities of CT surgeons. Clinical students disagreed significantly more than CT surgeons have personalities that are easy to get along with (μ=2.59 versus μ=2.93, P<0.008). Positive factors associated with CT surgery include CT surgery is a growing field that will continue to thrive, CT surgeons are leaders of their healthcare team, and CT surgeons are appropriately compensated for their work, with an overall mean of 3.85, 3.90, and 3.89, respectively. Within the positive factors, preclinical and clinical students had differing responses. Preclinical students agreed significantly more that CT surgery is a growing field that will continue to thrive (μ=3.97 versus μ=3.59, P<0.004). Preclinical students also agreed significantly more with the statement that CT surgeons are leaders in their healthcare team (μ=4.00 versus μ=3.68, P<0.012).

Student interest and exposure to CT surgery had the most differences between preclinical and clinical student responses. Preclinical students had significantly higher Likert ratings for the statement that interest group involvement will impact the electives they choose during their clinical years (μ=3.62 versus μ=3.10, P<0.008). Both preclinical and clinical students highly disagree that they have gained adequate exposure to CT surgery during their preclinical years (μ=1.73 versus μ=1.57). Preclinical students also had higher agreement ratings that exposure to CT surgery during preclinical years would further increase their interest when compared to clinical students (μ=4.12 versus μ=3.51, P<0.000). A similar response was elicited when asked if exposure during clinical years increased interest (μ=4.24 versus μ=3.76, P<0.010). Last, clinical students had significantly lower agreement ratings that they would be supported by their school to pursue CT surgery compared to preclinical students. (μ=2.45 versus μ=3.40, P<0.000).

## Discussion

A previous study by this group conducted a similar survey at a single osteopathic medical school. This single-institution study included similar questions asked in this survey, including what medical students’ specialty interests are, positive and negative factors associated with CT surgery, and factors that increased medical students’ interest in CT surgery and when those factors occurred [5]. This trial expands to a national scale while narrowing the focus to medical students interested in surgery and its subspecialties.

In accordance with our first trial, there is greater expressed interest in CT surgery from preclinical students than clinical students. Additionally, factors that deterred both preclinical and clinical students alike from CT surgery include lifestyle as a resident, lifestyle as an attending surgeon, lack of exposure to CT surgical fields, and lack of CT surgeon mentors. Both our first study and Grover et al. [5,13] demonstrated that lifestyle as a resident and attending surgeon seems to push students away from surgical specialties in general. This study suggests that even among students interested in surgery, CT surgery is not considered due to the perception that CT surgeons and trainees have a poor lifestyle. Furthermore, respondents noted that lack of exposure to CT surgery and lack of mentors deterred them from pursuing the specialty. Unfortunately, this is congruent with the TSMA study by Aranda-Michel et al. [12] and denotes the significant need for CT surgery mentors and increased exposure of medical students to the field.

**Figure 7:**
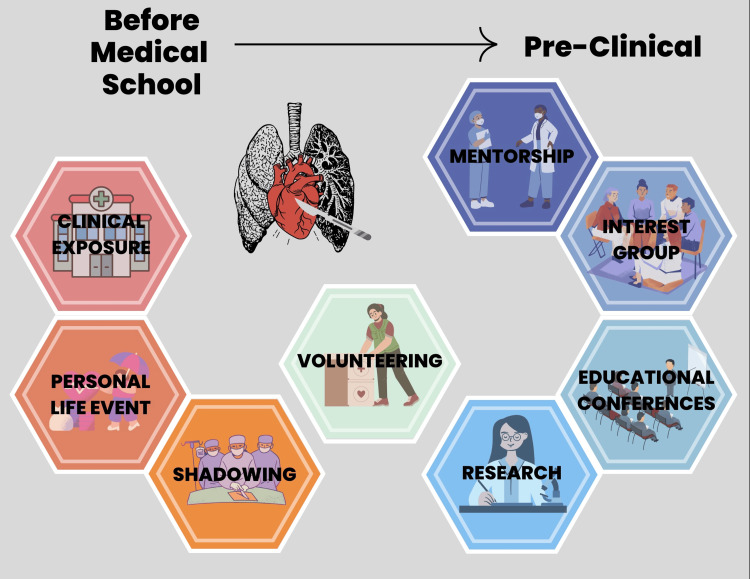
Avenues to cardiothoracic surgery Note: created with www.canva.com

In accordance with our first study, this survey assessed what factors increased a student’s interest in CT surgery and when they occurred during a student’s career. Clinical exposure and shadowing were the most selected factors overall. Similar to Aranda-Michel et al., these factors occurred most frequently before students entered medical school, indicating that most students who are interested in CT surgery developed this interest before they began their medical education (Figure 7) [5,12]. Additionally, Brennan and colleagues determined that developing shadowing programs for premedical students garnered interest in the field and offered opportunities to students without a family member physician to facilitate these connections [8].

This study found that during the preclinical years of medical school, students are impacted by interest group involvement, educational conferences, mentorship, and research while clinical students are most impacted by clinical rotations in CT surgery. In accordance with our second study, Davis et al., and Salna et al., interest groups seem to strongly influence preclinical students’ career pursuits, initiating or further strengthening an interest in CT surgery [10,11,14]. Our second trial also demonstrated a change in the initial negative perceptions of the field as students continued to attend more informative and uniquely designed events [11]. Building upon the second study, this current study demonstrates that educational conferences are impactful during the preclinical years. Conferences dedicated specifically to CT surgery and their scholarships dedicated to students are critical and impactful to the students who are able to attend earlier in medical school.

Though there may be a lack of exposure to CT surgery for medical students, a lack of awareness of accessible resources to facilitate exposure to the field significantly lowers the likelihood that students will utilize them. In almost all questions analyzing students’ knowledge of their school’s CT surgery alumni, training programs, and elective rotations, preclinical students were significantly less aware of available resources associated with their institution. Furthermore, our data demonstrated that there is a lack of interest groups dedicated to CT surgery. As previously stated, surgery interest group involvement proved impactful on preclinical students’ interest in CT surgery, showing that CT-specific interest groups could potentially prompt a similar increased interest in the field [11,14].

Even among students interested in surgery, there continues to be a common theme of negative perceptions associated with CT surgery, including work/life balance, personalities that are easy to get along with, and surgeons having time for their families [5,7,11]. While these findings reiterate the results of previous trials, this data is unique in that these are the perceptions specifically of students looking to pursue surgery, not the overall perception of medical students. This highlights additional barriers that must be faced when developing not only students’ interest in surgery but, more specifically, CT surgery. Consequently, it is imperative that the field finds ways to confront these negative perceptions held by this subgroup of medical students and ameliorate them. Furthermore, our data suggests that there is a need for CT surgery exposure during all four years of medical school as students do not feel they are adequately receiving exposure to the field at all. These nuances are worth consideration as areas of untapped potential for CT surgeons to interact with medical students earlier in their training when such interactions seem to be more impactful.

Finally, this study determined that clinical students feel significantly less supported to apply to a CT surgery residency program when compared to preclinical students. This posits the question as to why students may feel this shift in perceived support. Medical students who discover their interest in CT surgery at a later stage in their medical training may not feel they have adequate time to build an application to the caliber of someone who has been pursuing opportunities in CT surgery much earlier, ultimately deciding to pursue a specialty in which they feel they would be a more successful applicant. While the reasons for the lack of perceived support from their institutions are still to be further investigated, medical students can offset these effects by utilizing organizations such as ACOS-MSS and TSMA and the resources they offer to determine and develop their interest in CT surgery [12,15]. 

Limitations

This study has important limitations to consider. While this is a national survey, it encompasses only a subset of the medical students interested in surgery. The ACOS focuses on providing a platform for surgeons and medical students in a division of medicine where medical schools focus on producing primary care physicians. While this survey only assesses osteopathic medical students interested in surgery, we believe it may be applicable to all medical students at smaller medical schools that may not be attached to an academic medical center. Furthermore, this study was only conducted over one academic year and includes medical students who were impacted by the COVID-19 pandemic. This may cause variations in responses across classes and what may have drawn or deterred interest in CT surgery. Further research should be dedicated to a longitudinal trial that follows students throughout medical school and determines when and why medical students may lose interest in CT surgery. 

## Conclusions

While students state that they are deterred from CT surgery due to a lack of mentors and exposure to the field, those who are interested identify these exact same factors as reasons for gaining interest in the field, specifically before entering medical school. This identifies a considerable recruitment problem within the field that should be further emphasized to prospective CT surgery mentors. Future research should be dedicated to identifying when and what year medical students may lose interest in CT surgery, assessing the lack of support institutions may not be providing to students, and determining the most effective ways that the ACOS-MSS and TSMA can help their members feel prepared for and be successful in a career dedicated to the success of CT surgery.

## Appendices

Supplemental material 1

Interest in Cardiothoracic Surgery Among the American College of Osteopathic Surgeons' Medical Students:

Part 1: Demographics

1. What osteopathic medical school do you attend? (free response)

2. What community type does your osteopathic medical school reside in?

a. Rural

b. Suburban

c. Urban

3. Class Year

a. OMS-I

b. OMS-II

c. OMS-III

d. OMS-IV

4. Gender

a. Male

b. Female

c. Other

d. Prefer not to say

5. Race/Ethnicity

a. Caucasian

b. African-American

c. Latino or Hispanic

d. Asian

e. Native American

f. Native Hawaiian or Pacific Islander

g. Two or More

h. Other/Unknown

i. Prefer not to say

6. What is your age? (free response)

7. Did you start medical school immediately after completing your undergraduate degree?

a. Yes

b. No

8. Did you have a previous career before attending medical school?

a. Yes

b. No

9. What is your relationship status?

a. Single

b. Married/ Domestic Partnership

c. Divorced/ Widowed

d. Prefer not to say

10. To your knowledge, does your school have a Cardiothoracic Surgery Interest Group or Committee?

a. Yes

b. No

c. I am not sure

11. If so, did you or are you attending events hosted by the group?

a. Yes

b. No

Part 2: Cardiothoracic Surgery Questions

1. Please select the surgical subspecialties you are most interested in.

a. Cardiothoracic Surgery

b. Colon and Rectal Surgery

c. Critical Care Surgery

d. General Surgery

e. Neurological Surgery

f. Ophthalmology

g. Orthopedic Surgery

h. Otolaryngology

i. Pediatric Surgery

j. Plastic Surgery

k. Trauma Surgery

l. Urology

m. Vascular Surgery

n. Other

2. Please rate the below factors based on how much they have influenced your decision to consider not pursuing cardiothoracic surgery. If you have never experienced these factors, please do not select an option. (Likert 5-point scale) (Each option will include: 1. Strongly disagree, 2. Somewhat disagree, 3. Neither agree nor disagree, 4. Somewhat agree, 5. Strongly agree)

a. Workplace environment can be malignant

b. Lifestyle as a resident

c. Lifestyle as an attending surgeon

d. Personality of residents and surgeons encountered

e. Hours associated with residency training

f. Length of training to become an attending surgeon

g. Difficulty of residency training

h. Negative exposures during 3rd year clerkships

i. Subject matter is uninteresting

j. Competitiveness of COMLEX/USMLEs and class rank

k. Gender-specific reasons

l. Lack of exposure to cardiothoracic surgical fields

m. Previous work experiences

n. Lack of cardiothoracic surgeon mentors

o. Negative perception of cardiothoracic surgeons

p. Fellowship options after residency training

q. Previous or current research experiences

r. Compensation

s. Surgical Interest Group activities

t. Future job security

u. Reimbursement trends

v. Family member is a cardiothoracic surgeon

w. Never considered the option of becoming a cardiothoracic surgeon

x. Application of osteopathic principles

y. Other reasons (free response)

3. For the specialties that you are interested in, what started/continued your interest in the field? Please select all that apply and identify when you had this experience. (Each option will include: 1. Before medical school, 2. OMS-I and OMS-II, 3. OMS-III and OMS-IV, 4. N/A)

a. Clinical rotation

b. Clinical exposure

c. Educational conferences

d. Mentorship

e. Research

f. Shadowing

g. Events conducted by your school’s surgical interest group

h. Significant personal/life event

i. Volunteering

j. Other reasons (free response)

4. Please rate the following statements using the scale below. (Likert 5-point scale) (Each option will include: 1. Strongly disagree, 2. Somewhat disagree 3. Neither agree nor disagree, 4. Somewhat agree, 5. Strongly agree)

a. Cardiothoracic surgeons have a good work/life balance

b. Cardiothoracic surgeons have personalities that are easy to get along with

c. Cardiothoracic surgery is a growing field and will continue to thrive

d. Cardiothoracic surgeons are leaders of the healthcare team

e. Cardiothoracic surgeons are appropriately compensated for the work they perform

f. Cardiothoracic surgeons have time for their families

5. If you are interested in cardiothoracic surgery, what started/continued your interest? Please select all that apply and identify when you had this experience? (Each option will include: 1. Before medical school, 2. OMS-I and OMS-II, 3. OMS-III and OMS-IV, 4. N/A)

a. Clinical rotation

b. Clinical exposure

c. Educational conferences

d. Mentorship

e. Research

f. Shadowing

g. Events conducted by your school’s surgical interest group

h. Significant personal/life event

i. Volunteering

j. Other reasons (free response)

6. To your knowledge, has your school matched anyone into an integrated - 6-year program for Cardiothoracic Surgery?

a. Yes

b. No

c. I am not sure

7. To your knowledge, are you aware of any alumni who are currently completing a traditional cardiothoracic surgery residency?

a. Yes

b. No

c. I am not sure

8. To your knowledge, are you aware of any alumni at your school who are currently attending cardiothoracic surgeons?

a. Yes

b. No

c. I am not sure

9. To your knowledge, is there a cardiothoracic training program (Integrated or Traditional) at your institution?

a. Yes

b. No

c. I am not sure

10. To your knowledge, is there a cardiothoracic training program (Integrated or Traditional) located in the city or town of your osteopathic medical school?

a. Yes

b. No

c. I am not sure

11. To your knowledge, does your osteopathic medical school offer an elective rotation in cardiothoracic surgery?

a. Yes

b. No

c. I am not sure

12. Please rate the following statements using the scale below. (Likert 5-point scale) (Each option will include: 1. Strongly disagree, 2. Somewhat disagree 3. Neither agree nor disagree, 4. Somewhat agree, 5. Strongly agree)

a. My interest group involvement in OMS-I and OMS-II impacted which electives I chose or will choose to complete in OMS-III and OMS-OV.

b. I have gained adequate exposure to cardiothoracic surgery during OMS-I and OMS-II.

c. I have gained adequate exposure to cardiothoracic surgery during OMS-III and OMS-IV.

d. More OMS-I and OMS-II exposure to cardiothoracic surgery would increase my interest in the field.

e. More OMS-III and OMS-IV exposure to cardiothoracic surgery would increase my interest in the field.

f. I feel like I would be supported by my school if I choose to apply for a cardiothoracic surgery residency
